# Players in the silencing of retroviral DNAs in embryonic stem cells

**DOI:** 10.1186/1742-4690-10-S1-O13

**Published:** 2013-09-19

**Authors:** Sharon Schlesinger, Gary Wang, Andreia Lee, Stephen P Goff

**Affiliations:** 1 Columbia University, New York, NY, USA

## 

Embryonic stem (ES) cells potently repress retroviral infection through transcriptional silencing of the newly established proviral DNAs. We have characterized two distinct types of silencing in embryonic mouse cells infected by the Moloney murine leukemia virus (MLV): a highly efficient one targeted to the proline tRNA primer binding site (PBSpro) on the MLV genome, and a less efficient one occurring independently of the PBS. We determined that the PBS-specific silencing is mediated by TRIM28 (Kap-1), a known transcriptional silencer, tethered to the DNA by ZFP809, one of the dozens of zinc finger proteins encoded in the mammalian genome. At least two more components, HP1gamma and EBP1, are implicated in this silencing. We have also begun characterizing a non-PBS directed silencing complex acting more broadly on incoming viral DNAs in ES cells. We suggest that a major player in this process is Yin Yang1 (YY1), a GLI-Kruppel zinc finger protein, ubiquitously expressed and highly conserved between species. We show by ChIP that YY1 binds the LTR of both exogenous and endogenous retroviruses in ES cells. Deletion of the YY1 binding site from the MLV-LTR increased expression of the provirus at least four fold over that of the wild-type virus. Moreover, in YY1 KD cells the expression of the IAP (class II) endogenous retroviruses was remarkably upregulated.

